# 2494. Massachusetts HCV Treatment Report Card: Hepatitis C Virus Care Cascade for Women of Reproductive Age with Opioid Use Disorder, 2014-2020

**DOI:** 10.1093/ofid/ofad500.2112

**Published:** 2023-11-27

**Authors:** Rachel L Epstein, Ben Buzzee, Elizabeth Erdman, Alexander Walley, Benjamin P Linas

**Affiliations:** Boston University Chobanian and Avedisian School of Medicine, Boston, Massachusetts; Boston Medical Center, Boston, Massachusetts; Massachusetts Department of Public Health, Boston, Massachusetts; Boston University Chobanian and Avedisian School of Medicine, Boston, Massachusetts; Boston University School of Medicine/Boston Medical Center, Boston, MA

## Abstract

**Background:**

Hepatitis C virus (HCV) infection diagnoses continue to rise across the United States, particularly in reproductive age women. Few studies have characterized HCV testing, linkage to care, and treatment rates in this key population in the direct acting antiviral (DAA) era. We characterize the HCV care cascade among reproductive age women with opioid use disorder (OUD) and therefore at high risk for HCV infection in Massachusetts (MA), a state which removed all Medicaid HCV treatment restrictions in 2016.

**Methods:**

The MA Department of Public Health maintains the Public Health Data Warehouse (PHD), a linked longitudinal administrative records dataset. The PHD can link, at the individual level, substance use treatment datasets, the All-Payer’s Claims Database, vital statistics data, and HCV surveillance data. We included women 15-45 years old at high-risk for HCV infection due to OUD, which we defined as any OUD diagnosis using international classification of disease 9/10 codes, opioid-related overdose or death, or treatment for OUD between 2014-2020. We then calculated the number of women who achieved each HCV care cascade outcome overall and by calendar year: 1) tested for HCV (antibody or RNA), 2) confirmed HCV by case report, 3) linkage to HCV care (HCV as a primary encounter diagnosis), 4) HCV treatment (DAA prescription), and 5) testing for sustained virologic response ≥ 12 weeks after treatment end (SVR12).

**Results:**

Of 64,432 MA women at high risk for HCV, only 40,228 (64.4%) had HCV antibody or RNA testing during the study period (Figure 1). Of 9382 women with confirmed chronic HCV infection (23.3% of those tested), 6331 (67.5%) linked to care and 2711 (28.9%) had HCV treatment prescribed. Only 1682 (62.1% of those treated) had an SVR12 check. Case identification decreased over the study period and treatments increased until 2019 (Figure 2).

**Figure:**
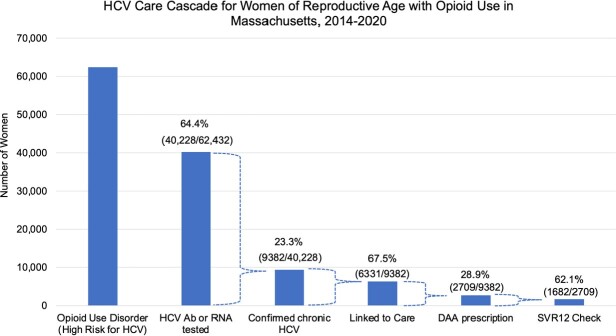

Hepatitis C Virus care cascade outcomes and percentages are conditional as noted: the proportion with confirmed chronic HCV (by case report between 2011-2020) is of those tested, the proportion linked to care and treated (DAA prescription) are of those with confirmed HCV, and SVR12 check (test for HCV cure at least 12 weeks after treatment end) is of those with chronic HCV prescribed a DAA. Abbreviations: HCV, Hepatitis C virus; Ab, antibody; RNA, ribonucleic acid; DAA, direct-acting antiviral; SVR, sustained virologic response

**Figure:**
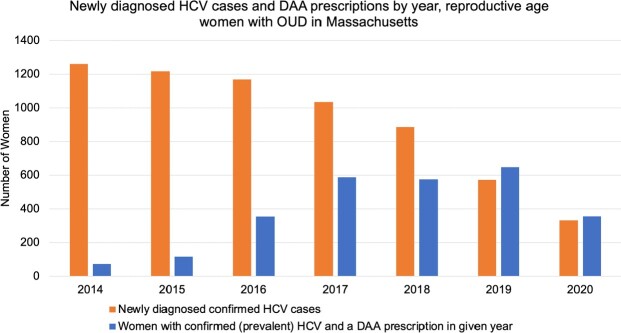

Abbreviations: HCV, hepatitis C virus; DAA, direct-acting antiviral; OUD, opioid use disorder

**Conclusion:**

Using an inclusive, statewide dataset we demonstrate an HCV treatment rate among reproductive age women with high-risk opioid use and confirmed HCV that is much higher than observed in other US studies, yet still < 50%. Importantly, nearly half of women at risk had not been tested for HCV. We have significant work to do to implement the 2020 universal testing recommendations to improve HCV testing and treatment rates to achieve HCV elimination goals.

**Disclosures:**

**All Authors**: No reported disclosures

